# A Clinical Prediction Model for Personalised Emergency Department Discharge Decisions for Residential Care Facility Residents Post-Fall

**DOI:** 10.3390/jpm15080332

**Published:** 2025-07-30

**Authors:** Gigi Guan, Kadison Michel, Charlie Corke, Geetha Ranmuthugala

**Affiliations:** 1 Department of Rural Health, Melbourne Medical School, The University of Melbourne, Shepparton, VIC 3630, Australia; granmuth@une.edu.au; 2 Critical Care Unit, Goulburn Valley Health, Shepparton, VIC 3630, Australia; kadison.michel1@gvhealth.org.au; 3 School of Medicine, Deakin University, Geelong, VIC 3220, Australia; charlie.corke@deakin.edu.au; 4 Critical Care Units, Barwon Health, Geelong, VIC 3220, Australia; 5 School of Rural Medicine, University of New England, Armidale, NSW 2350, Australia

**Keywords:** Advance Care Directive (ACD), detection, emergency department (ED), falls, prehospital care, nursing home, personalised medicine, prediction, residential aged care facilities (RACF)

## Abstract

**Introduction**: Falls are the leading cause of Emergency Department (ED) presentations among residents from residential aged care facilities (RACFs). While most current studies focus on post-fall evaluations and fall prevention, limited research has been conducted on decision-making in post-fall management. **Objective**: To develop and internally validate a model that can predict the likelihood of RACF residents being discharged from the ED after being presented for a fall. **Methods**: The study sample was obtained from a previous study conducted in Shepparton, Victoria, Australia. Consecutive samples were selected from January 2023 to November 2023. Participants aged 65 and over were included in this study. **Results**: A total of 261 fall presentations were initially identified. One patient with Australasian Triage Scale category 1 was excluded to avoid overfitting, leaving 260 presentations for analysis. Two logistic regression models were developed using prehospital and ED variables. The ED predictor model variables included duration of ED stay, injury severity, and the presence of an advance care directive (ACD). It demonstrated excellent discrimination (AUROC = 0.83; 95% CI: 0.79–0.89) compared to the prehospital model (AUROC = 0.77, 95% CI: 0.72–0.83). A simplified four-variable Discharge Eligibility after Fall in Elderly Residents (DEFER) score was derived from the prehospital model. The score achieved an AUROC of 0.76 (95% CI: 0.71–0.82). At a cut-off score of ≥5, the DEFER score exhibited a sensitivity of 79.7%, a specificity of 60.3%, a diagnostic odds ratio of 5.96, and a positive predictive value of 85.0%. **Conclusions**: The DEFER score is the first validated discharge prediction model for residents of RACFs who present to the ED after a fall. Importantly, the DEFER score advances personalised medicine in emergency care by integrating patient-specific factors, such as ACDs, to guide individualised discharge decisions for post-fall residents from RACFs.

## 1. Introduction

Falls are the most common reason for residents of residential aged care facilities (RACFs) to be transferred to the emergency department (ED) [1]. Residents who experience a fall are often transferred to the ED for assessment and monitoring. However, such transfers do not always reveal significant medical issues, and many individuals are discharged from the ED and returned to the RACF [2].

Routine transfer of post-fall patients to the ED can have significant consequences, including deterioration and delirium, thus worsening patient outcomes [3]. Additionally, such transfers increase healthcare utilisation and strain the healthcare system, leading to ED overcrowding and ambulance ramping [4]. The decision to transfer post-fall patients to the ED also arises from the absence of effective decision-making models that can guide the process regarding individuals who experience a fall in RACFs [5].

Existing models, such as the Morse Fall Scale and STRATIFY, are designed to identify the risk of falls and help prevent them [6]. They are not designed to facilitate decision-making about discharging post-fall patients from the ED. Models such as the Identification of Seniors at Risk and the Triage Risk Screening Tool assess the long-term vulnerability of post-fall patients but do not guide in deciding whether post-fall patients should remain in the ED or be discharged [7]. Furthermore, early warning system (EWS) scores that utilise physiological parameters, such as the National Early Warning System 2 (NEWS2) and the Modified Early Warning System (MEWS), lack specificity for this cohort of patients and often focus on short-term deterioration rather than facilitating discharge decisions [8,9].

To date, no validated model exists to facilitate decision-making regarding the discharge of RACF residents from the ED after they have been presented for a fall. Such models are essential, as they may assist RACF nursing staff members in managing post-fall patients within RACFs, as well as paramedics and ED staff members in making rapid discharge decisions. They may also reduce healthcare utilisation and admission-related complications and improve patient-centred care.

Furthermore, the current practice of transferring all post-fall patients to the ED does not provide personalised care in RACFs for geriatric patients [2,10]. Tailoring healthcare decisions to individual patient characteristics, is particularly crucial in managing fall-related injuries among RACF residents due to their diverse health profiles and care needs [2].

To address these needs, this study aimed to develop and internally validate a prediction model to determine the likelihood of RACF residents being discharged from the ED after presenting for a fall. This study also developed a simplified scoring system, known as the Discharge Eligibility after Fall in Elderly Residents (DEFER) score, to assess the likelihood of discharge for post-fall patients from the ED. The DEFER score aims to provide a tool that facilitates shared clinical decision-making among RACF nursing staff members, paramedics, and ED teams.

## 2. Methods

### 2.1. Design and Data Source

The study sample comprised the full dataset from a previous study conducted at Goulburn Valley Health (GVH) [10]. All patient information was obtained from GVH’s digital medical records (DMR). GVH is a rural hospital in Shepparton, 180 km north of Melbourne, Victoria, Australia. The study period was from 1 January 2023, to 19 November 2023.

During data collection, one case was excluded because of incomplete data. This exclusion was deemed to have a negligible impact on the study’s validity or findings, and there were no missing data in the analysis dataset. This secondary analysis was conducted with the same ethical approval as the original study, which was retrospectively approved by the GVH Human Research Ethics Committee in 2024 before data collection and analysis. The risk to patients is minimal as this study used a retrospective review of de-identified patient data, with a waiver of consent granted (Approval number: GVH 25-24).

### 2.2. Population

**Figure 1:**
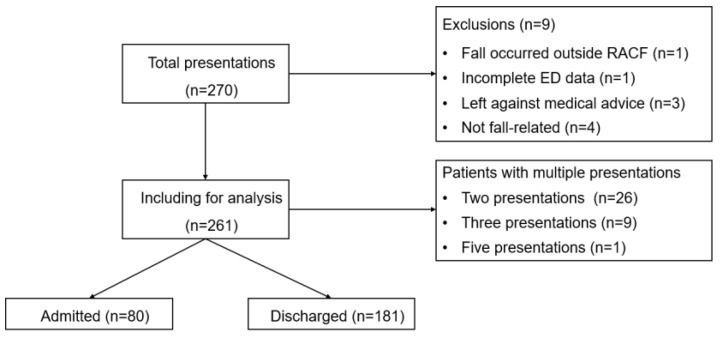
Flow chart of study sample selection.

This study collected data from individuals aged 65 or older who lived in RACFs and had presented to the ED due to fall-related presentations during the study period. Individuals were selected using triage notes, ICD-10 codes, and injury classifications. (Appendix A) This study excluded cases in which the patient died on arrival, the fall occurred outside the RACF (e.g., in the hospital or community), or an external cause (such as a motor vehicle accident) precipitated the fall. (Figure 1. Patient flow) A 10% random data audit showed an inter-rater agreement rate of 99.6%, indicating a robust data extraction process. The sample size was deemed to be sufficient based on the previous study.

### 2.3. Variables

The dependent variable was ED disposition, which was defined as whether the patient was admitted to the ED or discharged. If patients were admitted during an ED stay, the final disposition was from the ED or short-stay unit (an observation unit located within the ED). The key independent variables were injury severity, injury region, presence of an obvious injury, presence of pain, presence of an advance care directive (ACD), and duration of ED stay. In addition, the triage category was collected using the Australian Triage Scale (ATS, which ranges from 1 to 5, where 1 is the most urgent).

Independent variables included: Injury severity, which was classified as major or minor. Fractures, intracranial injuries, multiple injuries, and dislocations were considered major injuries, whereas superficial lacerations, muscle/tendon injuries, sprains/strains, and isolated open wounds were considered minor injuries. The injury region was classified as central or peripheral based on the anatomical region (Appendix B and Appendix C). An obvious injury was deemed to be present if there were any visible injuries, including haematoma, bruising, altered consciousness, inability to bear weight, and joint/bone angulation. The presence of pain was ascertained based on triage notes. The presence of an ACD was determined by reviewing triage notes, the ED discharge summary, and documents related to advance care planning. The duration of ED stay was automatically populated by the DMR and calculated from the time of ED presentation to the time of ED discharge or admission for inpatient care. As GVH is not a tertiary hospital, transfers out of GVH for admission or tertiary ED presentations were also included in the admissions count.

### 2.4. Data Analysis

All statistical analyses were conducted using Stata version 18 SE. For all inferential tests, statistical significance was set at α = 0.05. Patient characteristics, as described in the ED, were compared for model development.

### 2.5. Model Development and Selection Strategy

Using Transparent reporting of a multivariable prediction model for individual prognosis or diagnosis as a guide, this study constructed prehospital and ED models using the respective variables in each setting. Only one case had an ATS triage level 1; therefore, it was excluded from data analysis to avoid overfitting.

Due to the small sample size and to avoid overfitting, multiple statistical methods were used in the model development. Initially, multivariate logistic regression was conducted to exclude non-significant variables. Then, the selection of remaining variables was refined using multiple complementary methods, including backward, forward, and stepwise selection, as well as likelihood ratio tests (*p* < 0.05), to ensure parsimony and model robustness. LASSO regression, which applies regularisation to shrink noninformative coefficients, was also used. In addition, we limited the number of predictors relative to the number of events and performed ten-fold cross-validation to evaluate model stability and generalisability. These steps were explicitly undertaken to minimise overfitting given the modest sample size.

Furthermore, a block-wise nested model testing approach was used to assess the incremental contributions of added variable blocks to the model’s performance. For each modelling strategy, discrimination and model fit were compared using the area under the receiver operating characteristic curve (AUROC), the Akaike information criterion (AIC), and the Bayesian information criterion (BIC). The final model for each setting was selected based on discrimination, simplicity, and internal stability. In this study, AUROC values between 0.60 and 0.69, 0.70 and 0.79, 0.80 and 0.89, and 0.90 and 1.0 indicated poor, acceptable, good, and excellent discriminatory powers of the outcome, respectively [8].

### 2.6. Model Evaluation and Internal Validation

Model discrimination was assessed using AUROC with 95% confidence intervals (CIs). The DeLong test was used to compare the significance of the two models [11]. Model calibration was evaluated using the Hosmer–Lemeshow goodness-of-fit test and by visually inspecting calibration plots that compared predicted and observed discharge rates across deciles. The predictive accuracy was further assessed using the Brier score, which was decomposed into components of reliability, resolution, and uncertainty. Classification metrics based on a 0.5 threshold were used to evaluate model performance, including sensitivity, specificity, positive and negative likelihood ratios (LR^+^, LR^−^), and diagnostic odds ratios (DORs). Ten-fold cross-validation was conducted to estimate the internal validity of the models. Odds ratios (ORs) were monitored for consistency, and multicollinearity was assessed using variance inflation factors (VIFs) to ensure model stability. Model sensitivity, specificity, and positive and negative predictive value (PPV, NPV) were also reported. To reduce the risk of overfitting with the modest sample size, we performed internal validation using 1,000 bootstrap replications. Model performance (AUROC) from each resample was compared with the original sample to calculate optimism-corrected AUROC and 95% CI. Finally, clinical utility was examined using decision curve analysis (DCA), which quantified the net benefit across varying decision thresholds.

### 2.7. Derivation of the DEFER Score

After validating the prehospital model, the final prehospital logistic regression model was converted into a four-variable scoring system for its application in clinical practice. The variable selection process is the same as that for the ED/prehospital model selection, including backward, forward, stepwise selection, and LASSO regression. The predictor variables retained in the final prehospital model were converted into integer-point values by scaling and rounding the original logistic regression coefficients. Higher DEFER scores are intentionally aligned with a greater likelihood of safe discharge.

This transformation preserved each variable’s relative predictive contribution while enabling easy calculation in a clinical setting. It also led to the creation of the DEFER score, which ranges from 2 to 9. The point allocation reflects reduced clinical severity and fewer significant findings (e.g., minor injuries, absence of obvious injuries, or the presence of an ACD), which collectively indicate that the patient is more likely to be managed within RACF settings.

### 2.8. Evaluation of the Performance of the DEFER Score

The DEFER score was subjected to the same tests as the original ED/prehospital model to assess its discriminatory performance, calibration, and clinical utility. The AUROC, Brier score, Hosmer–Lemeshow goodness-of-fit, ten-fold cross-validation, DCA, and DOR were redetermined for the DEFER score. Although the Liu method identifies a cut-off point of 5.5, we selected a threshold of ≥5 for the DEFER score due to its clinical interpretability and superior predictive value in identifying patients suitable for discharge from the ED [12]. Consequently, the patients were divided into two groups based on the total score calculated from specific variables: low discharge-likelihood group (<5) and high discharge-likelihood group (≥5).

## 3. Results

A total of 261 RACF presentations occurred during the study period. 69.4% were discharged from ED (*n* = 181) and 30.6% were admitted for inpatient care (n = 80). The discharged patients were more likely to have ACD, minor injuries, peripheral injuries, and lower ATS triage levels (*p* < 0.05 for all variables). Other factors, such as the use of anticoagulants (*p* = 0.75) and computer tomography brain imaging (*p* = 0.14), did not vary significantly between the two groups (Table 1).

### 3.1. Model Development and Discriminative Performance

After removing one case with triage level 1, 260 presentations were analysed. The ED and prehospital models were developed using logistic regression and multiple variable selection processes. Using variables exclusively available at first clinical contact, the prehospital model retained four predictors: injury severity, presence of an obvious injury, presence of an ACD, and presence of pain. The ED model incorporated information available after in-hospital assessment and included three predictors: duration of ED stays (a post hoc predictor), injury severity, and presence of an ACD (Table 2).

**Figure 2:**
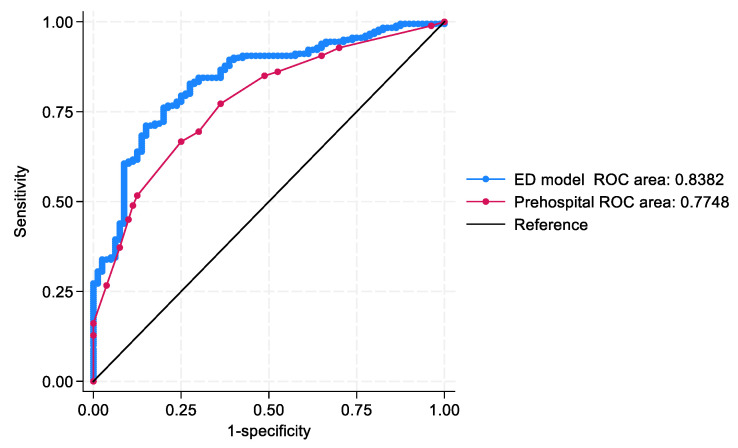
The area under the Receiver operating characteristic (AUROC) curve compares the ED-based model (Blue colour, AUROC 0.83) with the prehospital model (Red colour, AUROC 0.77) in predicting discharge. The ED model curve is above the prehospital model curve, indicating better discrimination.

The ED model (AUROC = 0.83; 95% CI: 0.79–0.89) and the prehospital model (AUROC = 0.77; 95% CI: 0.72–0.83) demonstrated acceptable to excellent discrimination. A comparative analysis using the DeLong test confirmed that the ED model significantly outperformed the prehospital model in distinguishing discharge outcomes (*p* = 0.04) (Figure 2).

### 3.2. Evaluation of Model Performance and Validation

The calibration of both models was acceptable. The Hosmer–Lemeshow goodness-of-fit test showed a non-significant result for both models, indicating the absence of miscalibration. Specifically, the ED model showed χ^2^ (8) = 12.21, *p* = 0.14, while the prehospital model showed χ^2^ (7) = 2.23, *p* = 0.95. Visual inspection of the calibration plots revealed close agreement between the predicted and observed probabilities in the ED model, whereas the prehospital model showed a modest overprediction (Appendix D).

According to the Brier scores, the predictive accuracy was better in the ED model (0.15) than in the prehospital model (0.17). The decomposition of this score showed greater resolution (0.06 vs. 0.04) and higher reliability (0.006 vs. 0.001) in the ED model, as well as equivalent uncertainty (0.21) across both models. Classification metrics based on a 0.5 threshold further highlighted the differences in the performance of the two models. The ED model showed a sensitivity of 90.6%, specificity of 56.3%, LR^+^ of 2.07, LR^−^ of 0.17, and DOR of 12.33. Meanwhile, the prehospital model showed a sensitivity of 86.1%, specificity of 47.5%, LR^+^ of 1.64, LR^−^ of 0.29, and DOR of 5.61.

Ten-fold cross-validation revealed good results for both models. The ED model exhibited a mean AUROC of 0.84, whereas the prehospital model showed a mean AUROC of 0.73. The predictor coefficients remained stable across folds, and no evidence of multicollinearity was detected. Internal validation using 1,000 non-parametric bootstrap methods showed the AUROC of the ED model was 0.84 (95% CI: 0.79–0.89; bootstrap standard error 0.03, *p* < 0.001), while the prehospital model had an AUROC of 0.78 (95% CI: 0.71–0.84; bootstrap standard error 0.0309, *p* < 0.001). The DCA demonstrated that both models provided greater net benefits than default strategies (‘treat all’ or ‘treat none’) across clinically relevant thresholds. The ED model performed marginally better than the prehospital model (Appendix E).

### 3.3. Derivation and Performance of the DEFER Score

The final prehospital model was translated into a simplified scoring system to support clinical implementation. This system comprised the DEFER score, which was calculated based on four predictors: injury severity, presence of an obvious injury, presence of an ACD, and presence of pain at the time of examination. Each assigned weighted-point value was based on the scaled regression coefficients.

The scoring algorithm and component weights are listed in Table 3. We assigned 2 points for a minor injury, 0 points for a major injury, 2 points for the absence of an obvious injury, 0 points for the presence of an obvious injury, 2 points for the absence of pain, 1 point for the presence of pain, 3 points for the presence of an ACD, and 1 point for the absence of an ACD. Consequently, the total score ranged from 2 to 9 (Table 3), and higher scores indicated an increased likelihood of ED discharge.

Patients were divided into two groups based on their discharge likelihood scores. The observed discharge rates for the low and high discharge-likelihood groups were 39.7% and 79.7%, respectively, reflecting clinically meaningful differences in discharge probability. For example, consider a 75-year-old RACF resident who presents after a fall with a minor lower limb laceration only (+2 points), no obvious external injuries such as fractures (+2 points), an existing advance care directive in place (+3 points), and reports pain on the laceration site only (+1 point). The total DEFER score would be 2 + 2 + 3 + 1 = 8 points. A score of 8 is ≥5, indicating a high likelihood of safe discharge, which means the patient can typically be managed in the RACF setting with appropriate follow-up rather than requiring hospital admission.

Post DEFER score testing, an AUROC of 0.76 (95% CI: 0.71–0.82) was achieved. The Hosmer–Lemeshow goodness-of-fit test showed non-significant results (χ^2^ (5) = 2.44, *p* = 0.79), indicating the absence of miscalibration between the predicted and observed discharge outcomes. With a cross-validated AUROC of 0.74 (95% CI: 0.68–0.81), the DEFER score showed robust internal validity and confirmed good discriminative performance across subsamples. The DEFER score also demonstrated good calibration with a cross-validated Brier score of 0.18. A calibration plot based on the decile grouping of the predicted probabilities revealed a close alignment between the predicted and observed discharge rates, indicating reliable probabilistic performance across risk strata (Appendix F). Furthermore, the DeLong test demonstrated that the DEFER score achieved comparable discrimination to the prehospital model (χ^2^ (1) = 1.38, *p* = 0.24).

Although Liu’s method identified a mathematically optimal cut-off score of 5.5, the DEFER score is an integer-based tool. Thus, a sensitivity analysis was conducted at these thresholds, including cut-off scores of ≥5 and ≥6, and using Liu’s method at a cut-off score of 5.5. At cut-off score ≥5, the DEFER score achieved a sensitivity of 79.7% (95% CI: 73.3–85.1%), specificity of 60.3% (95% CI: 47.7–72.0%), PPV of 85.0% (95% CI: 78.9–89.9%), and NPV of 51.2% (95% CI: 39.8–62.6%). This threshold provided the best balance for identifying patients suitable for discharge.

At cut-off score ≥6, sensitivity increased to 87.5% (95% CI: 79.9–93.0%) but specificity dropped to 44.6% (95% CI: 36.4–53.0%), with PPV declining to 54.4% (95% CI: 46.9–61.9%) and NPV improving to 82.5% (95% CI: 72.4–90.1%). This lower specificity results in more false positives at the cut-off score of ≥6. This indicates that patients who score high on the DEFER may still require hospital admission. In this context, false positives are less desirable than false negatives, as failing to provide ED-level care to patients who require inpatient care could result in missed treatment opportunities or delayed detection of deterioration.

The Liu method’s cut-off score of 5.5 achieved a specificity of 82% and a sensitivity of 54%, but PPV and NPV were not reported. This threshold would miss nearly half of the true discharges, limiting the tool’s usefulness. For the above reasons, the cut-off score ≥5 was selected as a clinically practical cut-off score, optimised as a rule-in tool that balances sensitivity and specificity while minimising the risk of inappropriate discharge. At the cut-off score ≥5, the DEFER score crosses a broad range of threshold probabilities (0.4–0.7) (Appendix G).

## 4. Discussion

### 4.1. Key Findings

The prediction model developed from ED and prehospital settings can be used to determine the likelihood of an RACF resident being discharged from the ED after a fall-related presentation. When comparing the two models, the prehospital model demonstrated a lower AUROC (0.77) than the ED model (0.83). Variables such as frailty, comorbidities, and cognitive status—known to influence fall outcomes—were omitted because they were not available in the dataset. This absence likely reflects that such assessments are not consistently documented in RACF records or prehospital settings, which may limit the generalisability of the prehospital model.

The newly developed and internally valid DEFER score is novel and innovative. The four-variable aggregate score ensures user friendliness while demonstrating good predictive power. The DEFER score supports more efficient resource allocation and improves patient-centred care by accurately identifying patients who may be rapidly discharged from the ED or managed within the RACF. Before this study, no validated tool existed to guide ED discharge decisions for RACF residents transferred to the ED after a fall.

The variables included in the DEFER score facilitate rapid decision-making in environments where access to physiological data is limited or absent. The discovery of ACD as a predictor for discharge decision-making highlights the importance of patients’ wishes in medical care. Incorporating patients’ wishes enables patient-centred care and may drive system-wide improvements in healthcare efficiency for RACF residents experiencing falls.

### 4.2. Clinical Utility of the DEFER Score

Unlike other EWS scores, such as NEWS2 or MEWS, the DEFER score uses non-physiological parameters [9]. The variables included in this score are often accessible during decision-making, making the DEFER score a pragmatic solution for discharge decision-making in settings where diagnostic infrastructure is unavailable or limited. This score may streamline ED disposition decisions and help prevent avoidable admissions, offering significant value to resource-constrained rural hospitals.

The DEFER score may be integrated into inter-professional communication to ensure the best outcomes for RACF residents who experience a fall. Integrating the handover process among RACF nursing staff members, paramedics, and the ED team can facilitate a shared decision-making process that aligns with patients’ wishes. When the DEFER score ≥5, it supports the decision to discharge; such patients may be safely cared for within the RACFs.

However, to manage residents within the RACF, facilities must ensure appropriate medical follow-up (for instance, by using telehealth consultations or medical reviews) and monitor the patients for delayed complications [13]. Implementing our model in practice would entail strengthening the care capabilities of RACFs and improving communication with ED and ambulance services.

In practice, a DEFER score of ≥5 indicates approximately an 80% chance that the patient can be discharged safely, whereas a score of <5 suggests caution and likely admission. However, integrating key clinical exclusions, such as unstable vital signs or concerning injuries, may enhance safety and sensitivity, especially for paramedics or RACF staff deciding whether an ED transfer is required. This is because the DEFER score was optimised for specificity and PPV and intended to function as a rule-in tool for discharge rather than as a broad screening instrument.

### 4.3. Other Factors Influencing Discharge

Discharge decisions, the management of patients within RACFs, and long-term fall outcomes are often complex in RACF settings [14]. Moreover, several complex conditions must be considered among RACF residents, such as existing comorbidities, frailty, cognitive changes, and, more importantly, post-fall physiological changes [15,16]. Thus, like any other EWS score, the DEFER score alone cannot predict post-fall outcomes in a real clinical context.

Variables in the DEFER score, such as pain and injury severity, may indirectly reflect a resident’s physiological reserve or frailty [17]. When in doubt, a lower score should be assigned, particularly in the presence of pain. ACDs often reflect care preferences linked to cognitive or functional decline [18]. While the DEFER score prioritises feasibility, essential in fast-paced or resource-limited settings, this focus limits its ability to address more complex cases.

Like all other predictive scores, the DEFER score should complement clinical judgement, not replace it. The DEFER score is designed not to reassure clinicians but to improve decision-making by highlighting cases where discharge is highly likely and further ED care may not be needed. Current post-fall protocols often reassure clinicians by over-investigating rather than improving patient outcomes by providing patient-centred care [19]. Importantly, the DEFER score is intended to be integrated within multidisciplinary workflows involving RACF staff, paramedics, and ED clinicians, rather than applied in isolation. It is designed to inform, not replace, clinical judgement—particularly in cases where abnormal vital signs, clinical instability, or other red-flag features are present. In such situations, clinical teams should prioritise comprehensive assessment and individualised decision-making, using the DEFER score as a supportive guide rather than a sole determinant.

While including ACDs in the DEFER score supports alignment with documented preferences, variability in ACD documentation and interpretation presents an ongoing challenge [18]. Documentation of ACDs can vary between RACFs, pre-hospital records, and ED settings, potentially limiting the reliability of this predictor. Strategies to address this include standardising ACD forms across services, implementing electronic health record integration so that ACDs are accessible in real time, and providing staff training on routine ACD verification and recording during transfers. Such measures could improve consistency and further enhance the applicability of the DEFER score across various settings. Clinicians should be aware that broader systemic or behavioural factors, including clinician risk tolerance, institutional policies, and family expectations, may influence discharge decisions in such models.

### 4.4. Advancing Personalised Medicine in Emergency Care with the DEFER Score

With the development of the DEFER score, along with recent advancements in personalised medicine, this study emphasises that the “one size fits all” approach should not be applied in healthcare.

Recognising that individuals, particularly geriatric patients, have differences in genetics, lifestyle, and, more importantly, individuals’ preferences, which all have an impact on health outcomes [20]. Standardised protocols often failed to address the diverse needs of patients, particularly patients from RACFs who had fallen. The DEFER score is a pioneering tool designed to predict the likelihood of safe discharge for these patients by incorporating non-physiological parameters, such as ACD. By integrating ACDs, the DEFER score ensures that discharge decisions align with patients’ values and wishes, fostering a patient-centred approach that respects individual preferences. This personalised strategy has the potential to reduce unnecessary hospitalisations, which can be costly and exacerbate health decline in frail elderly patients.

Current research has demonstrated that personalised geriatric medicine can reduce hospitalisation, which in turn, improves health outcomes [21]. Similarly, the DEFER score aims to optimise resource use and enhance patient outcomes by facilitating timely, individualised discharge decisions. The score’s simplicity and practicality make it particularly suited for the fast-paced ED environment, where time constraints and high patient volumes often hinder the implementation of personalised care. In addition, in resource-limited settings, such as prehospital and within RACFs, the DEFER score serves a crucial and valuable purpose in facilitating the decision-making process.

By providing a quick, straightforward method to incorporate patient-specific information, the DEFER score enables clinicians to make informed, efficient, and personalised decisions, addressing a critical gap in emergency care for vulnerable populations.

### 4.5. Implementation Pathway and Future Research

Future research should focus on external and prospective validation of the DEFER score in diverse settings. This includes other rural, regional, and metropolitan healthcare settings, particularly those with limited access to out-of-hospital care. Additionally, various state-wide and local post-fall protocols require interstate validation studies. This will help assess generalisability and refine the model’s performance. Prospective studies must also address other downstream factors of RACF outcomes, such as readmission, mortality, and patient or family satisfaction.

Integrating existing triage models, paramedic assessment tools, ED decision-making models, and the RACF post-fall screening protocols may enhance the implementation of the DEFER score. In the ED, the DEFER score may facilitate rapid triage decisions and assist junior clinicians and discharge planners by providing structured, evidence-informed support for complex discharge decisions. In prehospital settings, paramedics may use the DEFER score to facilitate more detailed clinical examinations and observations, thereby informing discharge decisions. In RACF settings, nursing staff members can apply the DEFER score after a fall and use the results to guide consultations with general practitioners and ambulance services.

The successful uptake of the DEFER score requires interprofessional education, alignment with existing protocols, and integration with the healthcare system. Future research should explore optimal ways for integrating the DEFER score into clinical workflows, such as incorporating it into ambulance assessment checklists or RACF nursing protocols. It should also measure outcomes, such as reduced transfer rates and patient safety.

## 5. Strengths and Limitations

The DEFER score is a novel and clinically useful model for predicting discharge outcomes among RACF residents presented to the ED after a fall. This score includes a minimal set of variables—four non-physiological factors—readily available at the bedside, thus facilitating rapid decision-making. Furthermore, the incorporation of ACDs in the score ensures patient-centred care. However, several limitations exist before the widespread application of the DEFER score.

First, this study’s inherent retrospective, single-centred design may have limited the generalisability of its findings. While the DEFER score performed well in a rural Australian cohort, external and prospective validation in metropolitan EDs and other rural regions will help determine whether the model is accurate, especially when there are different admission thresholds or RACF resources.

Second, the dataset included only fall-related presentations that were brought to the local ED. Data on patients managed within the RACF or bypassed the local ED was unavailable. Thus, introducing a selection bias may reduce the overall performance of the DEFER score.

Third, ‘safe discharge’ is a context-sensitive concept influenced by local factors, such as RACF staffing, the availability of medical reviews, risk-averse practices within RACFs, local admission norms, and diagnostic capacity. Consequently, the model’s recommendations may not translate uniformly across settings with different care infrastructures.

In addition, our analysis assumed that factors such as pain and obvious injuries were accurately documented. However, in practice, under-documentation may have affected the model’s performance. Nevertheless, our comprehensive data extraction process ensured that no missing data was present, which mitigates this concern.

Finally, despite rigorous model selection and validation, the small sample size may have led to overfitting of the model, as other important factors such as comorbidities, frailty, or cognitive status were not included in the subgroup analysis. As a result, the DEFER score may be less applicable in residents with more complex clinical profiles, such as patients with significant comorbidities, marked frailty, or cognitive impairment. In these cases, additional clinical assessment and multidisciplinary input remain essential, and the score should be interpreted with caution.

## 6. Conclusions

The prediction model developed from prehospital and ED settings effectively guides ED discharge decisions for RACF residents presented to the ED after a fall. Such models can also determine whether a patient can be managed within RACFs. Using four easily obtainable variables, the newly developed DEFER score has a good discriminative effect on decision-making. Notably, the inclusion of ACD ensures that discharge decisions are aligned with each patient’s unique preferences and values, thereby advancing personalised medicine in emergency care settings. This patient-centred approach is particularly crucial for elderly patients, who often have complex care needs and specific wishes regarding treatments, fostering respect for patients’ autonomy and enhancing care quality. However, prospective studies are needed to confirm these results. Future studies should focus on the external validation of the DEFER score and its integration into clinical workflows to maximise its impact on RACFs and policies. The broader implementation of the DEFER score has the potential to deliver system-level benefits, including reducing unnecessary hospital admissions, optimising the use of limited healthcare resources, and enhancing patient and family satisfaction with care.

## Figures and Tables

**Table 1:** Characteristics of ED presentations following falls in RACFs by discharge status (*n* = 261).

Characteristics of ED Presentations				
	Total	Discharged	Admitted	*p* Value
	**261**	**181 (69.4%)**	**80 (30.6%)**	
**Age, median (max-min)**	87 (66–101)	87 (68–101)	86 (66–101)	0.538 ^†^
**Prior medical review**	50	37 (74.0%)	13 (26.0%)	0.428
**First Nations status**	9	6 (66.7%)	3 (33.3%)	0.819
**Duration of ED stay (mean minutes, SD)**	629.8 (450.7)	485.1 (368.2)	956.9 (450.9)	**<0.001**
**Use of anticoagulant or antiplatelet agents**	156	107 (68.6%)	49 (31.4%)	0.746
**Female**	166	115 (69.3%)	51 (30.7%)	0.974
**Mode of Presentation,** ***n*** **(%)**				
**Ambulance**	250	172 (68.8%)	78 (31.2%)	0.359
**Private vehicle**	11	9 (81.82%)	2 (18.18%)	
**ATS,** ***n*** **(%)**				
**ATS-1 Immediate**	1	1 (100%)	0 (0%)	
**ATS-2 Time-Critical**	17	8 (47.1%)	9 (52.9%)	**0.028**
**ATS-3 Urgent**	216	149 (69.0%)	67 (31.0%)	
**ATS-4 Potential**	27	23 (85.2%)	4 (14.8%)	
**Injury Severity,** ***n*** **(%)**				
**Major**	132	73 (55.3%)	59 (44.7%)	**<0.001**
**Minor**	129	108 (83.7%)	21(16.3%)	
**Body Injury Region,** ***n*** **(%)**				
**Central**	180	48 (26.7%)	132 (73.3%)	**<0.001**
**Peripheral**	81	49 (60.5%)	32 (39.5%)	
**ACD,** ***n*** **(%)**				
**Presence of ACD**	115	91 (79.1%)	24 (20.9%)	**<0.001**
**Advised transfer to ED**	53	9 (17.0%)	44 (83.0%)	0.719 ^‡^
**Against transfer to ED**	15	3 (20.0%)	12 (80.0%)	
**ED Triage Notes,** ***n*** **(%)**				
**Fall**	257	177 (68.9%)	80 (31.1%)	0.316 ^‡^
**Obvious injuries**	140	85 (60.7%)	55 (39.3%)	**<0.001**
**Head strike**	71	52 (73.2%)	19 (26.8%)	0.405
**CTB,** ***n*** **(%)**				
**Had CTB**	192	138 (71.9%)	54 (28.1%)	0.140
**Normal CTB results**	180	133 (73.9%)	47 (26.1%)	**<0.001** ^‡^
**Abnormal CTB results**	11	4 (36.4%)	7 (63.6%)	

Bold text in the *p* values indicates *p* < 0.05. ^†^ Mann–Whitney U-test; ^‡^ Fisher’s exact test. All other *p*-values were calculated using the chi-square test. *p* < 0.05 was considered statistically significant. Abbreviations: ACD = Advance Care Directive; CTB = computerised tomography brain scan; ATS = Australasian Triage Scale.

**Table 2:** Multivariate logistic regression analysis of factors associated with emergency department discharge following fall-related presentations from residential aged care facilities (n = 260) *p* value in bold indicates *p* < 0.05.

Variable	Adjusted Odds Ratio	95% CI (Lower-Upper)	*p* Value
Presence of ACD	2.8877	**1.3773–6.0543**	**0.005**
ATS	2.6892	**1.0634–6.8008**	**0.037**
CTB performed	2.1835	0.9149–5.2114	0.078
Body injury region (Central vs. Peripheral)	1.5383	0.6699–3.5328	0.310
Sex (Male vs. Female)	1.2519	0.5914–2.6501	0.557
Fall was documented in the triage notes	1.1072	0.5694–2.1530	0.764
Age	1.0130	0.9637–1.0649	0.611
Prior medical review	0.9013	0.3597–2.2582	0.824
First Nations status	0.7013	0.2493–1.9728	0.501
Anticoagulant/antiplatelet use	0.6380	0.3079–1.3222	0.227
Head strike in triage notes	0.8394	0.3586–1.9644	0.686
Pain was documented in the triage notes	0.4802	0.2079–1.1091	0.086
ED duration (minutes)	0.9974	**0.9966–0.9983**	**<0.001**
Obvious injury	0.2393	**0.1022–0.5603**	**0.001**
Injury severity	0.2004	**0.0941–0.4270**	**<0.001**

Outcome variable: ED disposition (vs. admission). Abbreviations: ACD = Advance Care Directive; CTB = computerised tomography brain; CI = Confidence Interval; ATS = Australian Triage Scale. The model was adjusted for age, sex, injury severity, CTB findings, ACD presence, and other covariates as listed. Values represent adjusted odds ratios from a multivariate logistic regression model. Bold text in 95% CI (Lower-Upper) indicates aOR did not cross 1.

**Table 3:** Variables and points allocation in the DEFER score.

Variable	Condition	Points
Injury severity	Minor injury	+2
	Major injury	0
Obvious injury	No obvious injury	+2
	Obvious injury	0
Advance Care Directive	Present	+3
	Absent	+1
Pain on examination	Absent	+2
	Present	+1
Total DEFER Score (Range: 2 to 9)		
Interpretation	Score ≥5	Increased likelihood of safe discharge
	Score <5	Consider further evaluation or admission

## Data Availability

Data is available upon reasonable request to the corresponding author.

## Appendix A. Inclusion Criteria

1. ICD-10 with a fall-related presentation in either primary or secondary diagnosis.

• W00-W19: Falls.
• R29.6: Tendency to fall, not elsewhere classified.
• X59: Exposure to unspecified factor causing other and unspecified injury.
• R26: Abnormalities of gait and mobility.
• R42: Dizziness and giddiness.
• H81: Disorders of vestibular function.
• R55: Syncope and collapse.
• W25: Contact with sharp glass fall involving glass.

2. If, based on documentation from the ED notes, the injury’s cause was due to a fall.

## Appendix B. Injury Severity

Major Injuries:

• Fracture
• Intracranial Injury
• Multiple Injuries
• Dislocation
• Crushing Injury

Minor Injuries:

• Superficial
• Laceration
• Muscle/Tendon Injury
• Sprain/Strain
• Open Wound

## Appendix C. Body Injury Location (Central vs. Peripheral)

Based on the criteria below and anatomical location, the body injury locations were classified as central or peripheral. Patients with central and peripheral injuries were classified as having a central injury.

Central classification:

• Hip;
• Back, lower (includes loin);
• Face;
• Neck;
• Abdomen;
• Pelvis (includes anogenital, perineum);
• Thorax;
• Head.

Peripheral body region:

• Knee;
• Ankle;
• Leg, lower;
• Shoulder;
• Forearm;
• Hand (includes fingers);
• Elbow;
• Foot (includes toes);
• Wrist;
• Thigh;
• Upper arm

## References

[1] Wabe N., Seaman K.L., Nguyen A.D., Siette J., Raban M.Z., Hibbert P., Close J.C.T., Lord S.R., Westbrook J.I. (2022). Epidemiology of falls in 25 Australian residential aged care facilities: A retrospective longitudinal cohort study using routinely collected data. Int. J. Qual. Health Care.

[2] Gullick D., Islam M.R. (2023). Exploring avoidable presentations from residential aged care facilities to the emergency department of a large regional Australian hospital. Aust. J. Rural Health.

[3] Li K.Y., Gore J.L., Phelan E.A., Hall J., Gunaje N., Sabbatini A.K. (2025). Potentially avoidable emergency department transfers among Medicare beneficiaries. Am. J. Emerg. Med..

[4] Goldberg E.M., Marks S.J., Resnik L.J., Long S., Mellott H., Merchant R.C. (2020). Can an Emergency Department-Initiated Intervention Prevent Subsequent Falls and Health Care Use in Older Adults? A Randomized Controlled Trial. Ann. Emerg. Med..

[5] Gurung A., Sendall M.C., Barnard A. (2021). To transfer or not to transfer: Aged care nurses’ decision-making in transferring residents to the emergency department. Collegian.

[6] Strini V., Schiavolin R., Prendin A. (2021). Fall Risk Assessment Scales: A Systematic Literature Review. Nurs. Rep..

[7] Gretarsdottir E., Jonsdottir A.B., Sigurthorsdottir I., Gudmundsdottir E.E., Hjaltadottir I., Jakobsdottir I.B., Tomasson G., Jonsson P.V., Thorsteinsdottir T. (2021). Patients in need of comprehensive geriatric assessment: The utility of the InterRAI emergency department screener. Int. Emerg. Nurs..

[8] Guan G., Lee C.M.Y., Begg S., Crombie A., Mnatzaganian G. (2024). Performance of 21 Early Warning System scores in predicting in-hospital deterioration among undifferentiated admitted patients managed by ambulance services. Emerg. Med. J..

[9] Guan G., Lee C.M.Y., Begg S., Crombie A., Mnatzaganian G. (2022). The use of early warning system scores in prehospital and emergency department settings to predict clinical deterioration: A systematic review and meta-analysis. PLoS ONE.

[10] Guan G., Ranmuthugala G., Michel K., Corke C. (2025). Factors Associated with Emergency Department Discharge After Falls in Residential Aged Care Facilities: A Rural Australian Observational Study. J. Clin. Med..

[11] DeLong E.R., DeLong D.M., Clarke-Pearson D.L. (1988). Comparing the areas under two or more correlated receiver operating characteristic curves: A nonparametric approach. Biometrics.

[12] Liu H., Tang Y., Zhang H.H. (2009). A new chi-square approximation to the distribution of non-negative definite quadratic forms in non-central normal variables. Comput. Stat. Data Anal..

[13] Amadoru S., Rayner J.A., Joseph R., Yates P. (2018). Factors influencing decision-making processes for unwell residents in residential aged care: Hospital transfer or Residential InReach referral?. Australas. J. Ageing.

[14] Rameli P.M., Rajendran N. (2022). Outcomes of complex discharge planning in older adults with complex needs: A scoping review. J. Int. Med. Res..

[15] Wilson N., Hilmer S., March L., Cameron I., Lord S., Mason R., Sambrook P. (2011). Physical functioning measures and risk of falling in older people living in residential aged care facilities. Ther. Adv. Musculoskelet. Dis..

[16] Lee C.-H., Chen Y.-A., Yang C.-M., Huang K.-H., Tsai T.-H., Chang Y., Shieh S.-H. (2024). Risk Factors Associated with Unplanned Hospitalization Among Long-Term Care Facility Residents: A Retrospective Study in Central Taiwan. Healthcare.

[17] Pecheva M., Phillips M., Hull P., Carrothers A O.L.R., Queally J.M. (2020). The impact of frailty in major trauma in older patients. Injury.

[18] Bryant J., Sellars M., Sinclair C., Detering K., Buck K., Waller A., White B., Nolte L. (2021). Inadequate completion of advance care directives by individuals with dementia: National audit of health and aged care facilities. BMJ Support. Palliat. Care.

[19] Lee C., Beavers J., Pham J., Hackett L., Miller J., Buntine P. (2022). Impact of the Canadian CT head rule supplemented by the original published minimum inclusion criteria to assist emergency department clinicians’ assessment of patients presenting post fall from residential aged care: A retrospective audit. BMC Geriatr..

[20] Australian Institute of Health and Welfare (2024). The ongoing challenge of chronic conditions in Australia. Australia’s Health 2024: Data Insights.

[21] Kotsani M., Kravvariti E., Avgerinou C., Panagiotakis S., Bograkou Tzanetakou K., Antoniadou E., Karamanof G., Karampeazis A., Koutsouri A., Panagiotopoulou K. (2021). The Relevance and Added Value of Geriatric Medicine (GM): Introducing GM to Non-Geriatricians. J. Clin. Med..

